# Renin-Angiotensin System Blockers Protect Pancreatic Islets against Diet-Induced Obesity and Insulin Resistance in Mice

**DOI:** 10.1371/journal.pone.0067192

**Published:** 2013-07-22

**Authors:** Eliete Dalla Corte Frantz, Camila Crespo-Mascarenhas, Andre Rodrigues C. Barreto-Vianna, Marcia Barbosa Aguila, Carlos Alberto Mandarim-de-Lacerda

**Affiliations:** Laboratory of Morphometry, Metabolism and Cardiovascular Disease, Biomedical Center, Institute of Biology, State University of Rio de Janeiro, Rio de Janeiro, Rio de Janeiro, Brazil; Broad Institute of Harvard and MIT, United States of America

## Abstract

**Background:**

The associations between obesity, hypertension and diabetes are well established, and the renin-angiotensin system (RAS) may provide a link among them. The effect of RAS inhibition on type 2 diabetes is still unclear; however, RAS seems to play an important role in the regulation of the pancreas and glucose intolerance of mice fed high-fat (HF) diet.

**Methods:**

C57BL/6 mice fed a HF diet (8 weeks) were treated with aliskiren (50 mg/kg/day), enalapril (30 mg/kg/day) or losartan (10 mg/kg/day) for 6 weeks, and the protective effects were extensively compared among groups by morphometry, stereological tools, immunostaining, Western blotting and hormonal analysis.

**Results:**

All RAS inhibitors significantly attenuated the increased blood pressure in mice fed a HF diet. Treatment with enalapril, but not aliskiren or losartan, significantly attenuated body mass (BM) gain, glucose intolerance and insulin resistance, improved the alpha and beta cell mass and prevented the reduction of plasma adiponectin. Furthermore, enalapril treatment improved the protein expression of the pancreatic islet Pdx1, GLUT2, ACE2 and *Mas* receptors. Losartan treatment showed the greatest AT2R expression.

**Conclusion:**

Our findings indicate that ACE inhibition with enalapril attenuated several of the deleterious effects of the HF diet. In summary, enalapril appears to be responsible for the normalization of islet morphology and function, of alpha and beta cell mass and of Pdx1 and GLUT2 expression. These protective effects of enalapril were attributed, primarily, to the reduction in body mass gain and food intake and the enhancement of the ACE2/Ang (1-7) /*Mas* receptor axis and adiponectin levels.

## Introduction

Obesity is strongly associated with both type 2 diabetes mellitus (T2DM) and hypertension, a combination that has become a major public health problem because of its epidemic proportions worldwide [1]. The renin-angiotensin system (RAS) is overexpressed when associated with obesity and its comorbidities and has emerged as an important target for pharmacological treatment [2,3].

Classically, the RAS is known for its role in body fluid and cardiovascular homeostasis. The RAS consists primarily of an enzymatic cascade through which angiotensinogen is converted to angiotensin (Ang) I, which is then converted to Ang II, through the action of renin and the angiotensin-converting enzyme (ACE) [4]. ACE also inactivates bradykinin [5], and Ang II mediates its specific functions via type 1 and type 2 receptors, i.e., AT1R and AT2R. Most of these functions are mediated by AT1R, including the potent vasoconstriction, proinflammatory, pro-oxidative, proliferative and hypertrophic effects. Moreover, advances in cell and molecular biology have allowed the recognition of other active elements of the RAS metabolism. Ang (1-7) may be formed primarily from Ang II (directly) and Ang I (indirectly) by the action of ACE 2, a homolog of ACE [6]. Through its G-protein-coupled receptor *Mas*, Ang (1-7) induces responses in opposition to those of Ang II, including vasodilation, antihypertrophic effects, and antiproliferative properties [7,8].

Over the past few years, RAS components have been found in almost every tissue, including the heart, blood vessels, kidney, brain, pancreas, adipose tissue and skeletal muscles [2]. Furthermore, a large body of evidence indicates that RAS activation has been closely correlated to both insulin resistance and beta cell dysfunction [9]. The mechanism behind this deleterious effect appears to be related to the negative regulation, exerted by Ang II through AT1R, of several steps of the insulin signaling cascade [10]. In addition, hyperglycemia increases the expression of RAS components in pancreatic islets, which leads to insulin secretion modulation in beta cells, decreased adiponectin, impaired insulin sensitivity in target tissues [11], inhibited GLUT4 translocation and increased levels of reactive oxygen species, inflammation, and ectopic fat storage [12].

The increase in the ACE2/Ang (1-7) /*Mas* receptor axis could be associated with diminished insulin resistance by inducing the activation of insulin signaling pathways and counteracting the inhibitory effects of ACE/Ang II/AT1R [7]. ACE2 gene therapy improves glycemic control in diabetic mice through a mechanism mediated by the Ang (1-7) /*Mas* receptor because of its proven ability to potentiate the action of bradykinin [13]. There is evidence that bradykinin itself may have an effect on enhancing insulin action and signaling [14]. Moreover, it is remarkable to note that, together with results from the beta cell injury, stands out the key role of the Pdx1(pancreatic-duodenal homeobox 1) in prenatal development of the pancreas, as well as the postnatal maintenance of the insulin production, and the transcriptional expression of GLUT (glucose transporter) 2 [15–17].

The present study aimed to compare the effect of blockades, using a direct renin inhibitor, an ACE inhibitor, and an AT1R antagonist, at different points in the RAS on glucose intolerance and pancreatic injury in a mice model of insulin resistance and obesity.

## Materials and Methods

### Animals and diet

Male C57BL/6 mice (12 weeks old) were maintained on a 12 h light/dark cycle (light on at 1 a.m.; light off at 1 p.m.), in a humidity- (60 ± 10%) and temperature- (21 ± 2 °C) controlled room. Animal care and procedures were in accordance with the conventional guidelines for experimentation with animals (National Institutes of Health Publication No. 85-23, revised in 1996) and were approved by the Animal Ethics Committee of the State University of Rio de Janeiro (Protocol Number CEA/21/2011).

The mice were fed *ad libitum* a standard chow (SC, n=15) diet (14% protein, 10% fat, and 76% carbohydrates, total energy 15 kJ/g) or a high-fat (HF, n=60) diet (14% protein, 50% fat and 36% carbohydrates, total energy 21 kJ/g). The diets were manufactured by PragSolucoes (Jau, Sao Paulo, Brazil) and were consistent with the recommendations of the American Institute of Nutrition (AIN 93M) [18]. After eight weeks of diet, the animals fed HF chow were randomly allocated into four groups, and each group received one of the following treatments over six weeks (the drugs were mixed into the diets, as follow):

a) HF group (n=15), untreated;
b) HF-A group (n=15), HF diet treated with aliskiren (50 mg/kg/day), Rasilez, Novartis;
c) HF-E group (n=15), HF diet treated with enalapril maleate (30 mg/kg/day), Renitec, Merck;
d) HF-L group (n=15), HF diet treated with losartan (10 mg/kg/day), Cozaar, Merck.

Both the SC and HF groups continued to have free access to their diet during the entire experimental period (eight weeks plus six weeks). Fresh chow was provided daily, and their food intake was evaluated. Energy intake was measured as the product of food consumption by the energy content of the diet. The body mass (BM) of the animals was measured weekly.

### Blood pressure

The animals were trained for two weeks in constraint conditions before the measurement of the blood pressure (BP) to minimize their stress. Twice a month, systolic BP was measured by tail-cuff plethysmography in conscious mice (Letica LE 5100; Panlab, Barcelona, Spain).

### Glucose and insulin tolerance

An oral glucose tolerance test (OGTT) was made after 6 h of food deprivation (1 a.m. -7 a.m.). Glucose (1.0 g/kg) was administered by orogastric gavage, and caudal vein blood samples were collected before and at 15, 30, 60 and 120 min after glucose administration. Blood glucose concentrations were measured using an Accu-Chek Go glucometer (Roche Diagnostics).

An intraperitoneal insulin tolerance test (IPITT) was performed after 4 h of food deprivation (1 a.m. -5 a.m.). Insulin (0.5 U/kg, Humalog Insulin Lispro, Lilly) was administered intraperitoneally, and caudal vein blood samples were collected before and at 15, 30, 60, 120 min after the injection. Blood glucose concentrations were measured using an Accu-Chek Go glucometer (Roche Diagnostics).

The area under the curve (AUC) was calculated for OGTT and IPITT from 0 to 120 minutes using the trapezoid rule (GraphPad Prism version 6.02 for Windows, GraphPad Software, La Jolla, CA, USA) to assess glucose intolerance and insulin sensitivity, respectively.

### Tissue extraction

After 14 weeks on the diet (including the 6 weeks of treatment), the animals were fasted overnight (food-deprived from 01:00 to 07:00 h) and then deeply anaesthetized (sodium pentobarbital, 150 mg/kg intraperitoneal). Blood samples were collected, and the plasma was separated by centrifugation (120 g for 15 min), used to measure fasting plasma glucose, and stored at -20 ºC until further analyses could be performed. The pancreas was completely dissected (n=6), or the islets were isolated by collagenase digestion (n=9). When dissected, the pancreas was weighed and then rapidly fixed in freshly prepared fixative solution [4% (w/v) formaldehyde and 0.1 M phosphate buffer, pH 7.2] for analysis by light microscopy (n=6).

### Metabolic data

The plasma concentrations of insulin and glucagon were evaluated using the Milliplex mouse metabolic hormone panel kit NMHMAG-44K, while the concentrations of adiponectin were evaluated with MADPK-71K-1ADPN with Luminex xMAP equipment (Millipore, Billerica, MA, USA). The homeostasis model assessment of insulin resistance (HOMA-IR) index, a simple assessment of insulin sensitivity, was calculated by the following formula: [fasting plasma glucose (mmol/L) × insulin (µUI/L)]/22.5 [19].

### Pancreas

#### Islet morphometry

Fixed pancreas samples were embedded in Paraplast plus (Sigma Aldrich, St. Louis, USA), sectioned into 5-μm-thick sections, and stained with hematoxylin and eosin. From the digital images of the pancreatic slides (TIFF format, 36-bit color, 1280×1024 pixels, LC Evolution camera and Olympus BX51 microscope), the smallest and largest diameters from each islet were measured to calculate the mean diameter (Image-Pro Plus version 7.01, Media Cybernetics, Silver Spring, MD, USA). At least 150 islets per group were measured, as described previously [20].

Next, the pancreatic fat density was estimated by a 36 test point test (P_T_) system superimposed on the tissue image by STEPanizer [21], as Vv[fat] = Pp/P_T_, where Pp represents the points that fall on fat cells in a sample of ten random fields per animal. 

#### Immunohistochemistry

For glucagon and insulin immunohistochemistry, deparaffinized and hydrated tissue sections were treated with citrate buffer, pH 6.0, at 60 °C for antigen retrieval and then with a 3% hydrogen peroxide solution in methanol to block endogenous peroxidase activity. The pancreas sections were incubated overnight with rabbit anti-glucagon (A0565, Dako; 1:100), or guinea pig anti-insulin (A0564, Dako; 1:100), followed by incubation with biotinylated secondary antibodies and streptavidinperoxidase conjugates (HistostainPlus Kit, Invitrogen, CA, USA). The sections were washed in PBS, revealed with liquid diaminobenzidine (HistostainPlus Kit, Invitrogen, CA, USA), and counterstained with hematoxylin.

#### Alpha and Beta cell masses

The islet volume density (Vv[islet]) and islet mass (M[islet]): Vv[islet] were estimated by point-counting using the ratio of the number of points that fell upon the pancreatic islet (Pp[islet]) and the total number of test-points in a test system made up of 36 test-points (P_T_): Vv[islet] = Pp[islet]/PT (%), assessed by STEPanizer [21]. Subsequently, M[islet] was obtained by multiplying the islet volume density by the pancreatic mass.

Alpha cell volume density (Vv[alpha cell]) and alpha cell mass (M[alpha cell]): Vv[alpha cell] were estimated using the glucagon-positive areas of the islets and was expressed as a percentage of the islet (Image-Pro Plus version 7.01, Media Cybernetics, Silver Spring, MD, USA). Thus, alpha cell mass was estimated as the product of Vv[alpha cell] and M[islet] [20].

Beta cell volume density (Vv[beta cell]) and beta cell mass (M[beta cell]): Vv[beta cell] were estimated by image analysis using the density threshold selection tool applied to islets with insulin-positive areas. Vv[beta cell] was expressed as a percentage of the islets (Image-Pro Plus version 7.01, Media Cybernetics, Silver Spring, MD, USA). Thus, the beta cell mass was estimated as the product of Vv[beta cell] and M[islet] [22].

#### Islet isolation

Islets were isolated by the collagenase method [23]. Briefly, the pancreas was cannulized via the bile duct and inflated *in situ* with cold Hanks’ solution (supplemented with foetal bovine serum 1 mg/mL) containing 0.8 mg/mL collagenase (C9263, Sigma Aldrich, St. Louis, USA). The pancreas was removed, placed into a tube and incubated in a 37 ºC water bath for 15 min to allow the digestion of the exocrine tissue. Afterwards, the tubes were vigorously shaken for approximately 15 seconds. The collagenase digestion was terminated by the addition of cold Hanks’ solution. The digest was then washed three times by filling the vial with Hanks’ solution. The islets were collected manually under a stereomicroscope (Luxeo 4D Stereozoom Microscope, Labomed, CA, USA) with a Pasteur pipette and immediately homogenized in extraction buffer (urea, 7 M; EDTA, 5 mM; Triton X-100, 1%; protease and phosphatase inhibitors).

### Western blotting analysis

The isolated pancreatic islet homogenate was centrifuged at 11,000 rpm at 4 ºC for 10 min, and the supernatant was collected. The lysate protein concentration was determined using a BCA protein assay kit (Thermo Scientific, Rockford, IL, USA). After denaturation, proteins were separated by polyacrylamide gel electrophoresis (SDS-PAGE) and transferred to a nitrocellulose membrane (Amersham Biosciences, Piscataway, NJ, USA). The membrane was then blocked by incubation in 6% (w/v) non-fat dry milk in TBS-T Tris-buffered saline [20 mmol/L Tris/HCl (pH 7.4) and 500 mmol/L NaCl] and sequentially incubated overnight at 4°C with the following primary antibodies: Pdx1 (anti-rabbit, AB3503; Chemicon; 1:1000), GLUT2 (anti-rabbit, 07-1402; Millipore; 1:1000), Renin (anti-mouse, SC137252; Santa Cruz Biotechnology; 1:1000), ACE (anti-rabbit, ab11734; Abcam; 1:500), AT1R (anti-rabbit, SC579; Santa Cruz Biotechnology; 1:1000), AT2R (anti-goat, SC48452; Santa Cruz Biotechnology; 1:1000), ACE 2 (anti-rabbit, ab108252; Abcam; 1:500) and *Mas* receptor (anti-rabbit, SC135063; Santa Cruz Biotechnology; 1:1000). Following incubation with the primary antibody, the membrane was incubated with the secondary antibody for 1 h at room temperature. The membrane was developed using ECL western blotting detection reagents, and images of the blot were obtained with Bio-Rad’s Molecular Imaging ChemiDoc XRS Systems (Bio-Rad, Hercules, CA, USA). The intensity of the chemiluminescent bands was quantified using ImageJ software, version 1.44 (NIH, imagej.nih.gov/ij, USA). The blots were stripped and reprobed for beta actin (anti-mouse, SC81178; Santa Cruz Biotechnology; 1:1000) as a loading control to normalize the blot data.

### Statistical analysis

The data were tested for normality and homoscedasticity of the variances. The differences among the groups were tested by one-way analysis of variance (ANOVA), followed by the Holm-Sidak post-hoc test. In all cases, *P*<0.05 was considered statistically significant (GraphPad Prism version 6.02 for Windows, GraphPad Software, La Jolla, CA, USA).

## Results

### Body mass and food behavior

**Figure 1:**
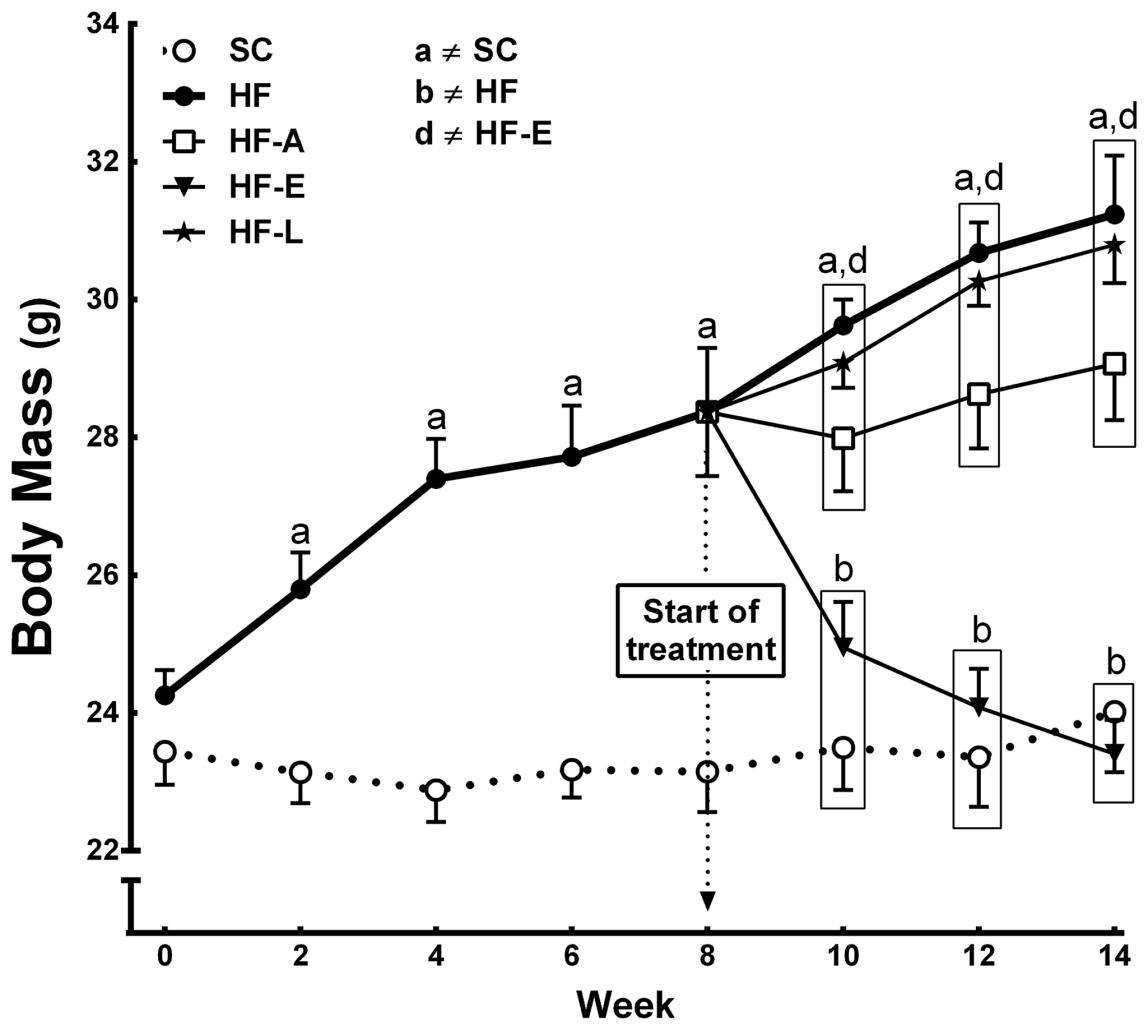
BM evolution over the course of the experimental period. Weeks 1–8 correspond to the period of the induction of obesity and insulin resistance, and weeks 8–14 correspond to the treatment phase. Values are means ± SEM, n=15. Significant differences between the groups are indicated using symbols (*P*<0.05), as determined by a one-way ANOVA and a post-hoc Holm-Sidak test: a ≠ SC; b ≠ HF; c ≠ HF-A, and d ≠ HF-E.

The animals started the study with no significant difference in their initial BM (Figure 1). After two weeks on their respective diets, the HF group demonstrated a higher BM than the SC group (+17%, *P*<0.001), and the difference in BM continued to increase up +31% in the HF group compared to SC group at eight weeks of diet consumption (*P*<0.0001). After six weeks of treatment, HF-E group showed a significant decrease in BM, 25% less than the HF group (*P*<0.0001). However, the HF-A and HF-L groups continued to be heavier than the SC group (+21% and +28%, respectively, *P*<0.0001) and the HF-E group (+24% and +31%, respectively, *P*<0.0001) (Figure 1).

The average weekly food intake was constant in each group over the period of six weeks of treatment, but the food intake was diminished in the HF-E group compared to the other groups (*P*<0.05). The energy intake was greater in the HF group than in the SC group (+35%; *P*<0.01), as indicated by calculating the energy provided by the diets based on their energy density (Table 1). Compared to the mice in the SC group after six weeks of treatment, the mice in the HF-A group had increased their energy intake by 31% (*P*<0.05), and the mice in the HF-L group had increased their energy intake by 44% (*P*<0.01). In the six-week treatment period, the mice in the HF-E group decreased their energy intake by 25% compared to the mice in the HF group (*P*<0.05), by 23% compared to the mice in the HF-A group (*P*<0.05), and by 30% compared to the mice in the HF-L group (*P*<0.01) (Table 1).

**Table 1:** Food behavior, carbohydrate metabolism and hormones.

**Data**	**SC**	**HF**	**HF-A**	**HF-E**	**HF-L**
**Food behavior**					
Food intake (g/week per mouse)	15.6 ± 0.6	16.0 ± 0.9	15.6 ± 0.9	12.0 ± 0.5^[a,b,c]^	17.1 ± 0.5^[d]^
Energy intake (kJ/week per mouse)	248.0 ± 9.9	334.8 ± 19.3^[a]^	325.8 ± 19.0^[a]^	251.2 ± 9.8^[b,c]^	357.4 ± 10.6^[a,d]^
**Carbohydrate metabolism**					
Glucose (mg/dL)	126.1 ± 8.3	168.8 ± 7.1^[a]^	153.8 ± 5.8^[a]^	133.2 ± 4.3^[b]^	153.0 ± 5.5^[a]^
HOMA-IR	5.3 ± 0.7	13.8 ± 1.5^[a]^	10.2 ± 1.0^[a]^	7.3 ± 0.5^[b]^	10.4 ± 0.7^[a]^
**Hormones**					
Insulin (µUI/L)	15.2 ± 1.6	30.8 ± 2.6^[a]^	23.6 ± 1.9^[a]^	21.8 ± 1.6^[b]^	26.4 ± 2.0^[a]^
Glucagon (pg/mL)	18.8 ± 4.1	42.3 ± 3.7^[a]^	32.8 ± 4.0^[a]^	24.3 ± 2.7^[b]^	39.0 ± 3.6^[a,d]^
Adiponectin (µg/mL)	17.6 ± 1.0	10.1 ± 0.5^[a]^	12.0 ± 0.4^[a]^	17.3 ± 0.7^[b,c]^	13.1 ± 1.0^[a,d]^

Values represent means ± SEM, n=8 per group. P<0.05 when compared with the SC group [a], HF group [b], HF-A group [c] and HF-E group [d] (one-way ANOVA and post-hoc Holm-Sidak test).

**Figure 2:**
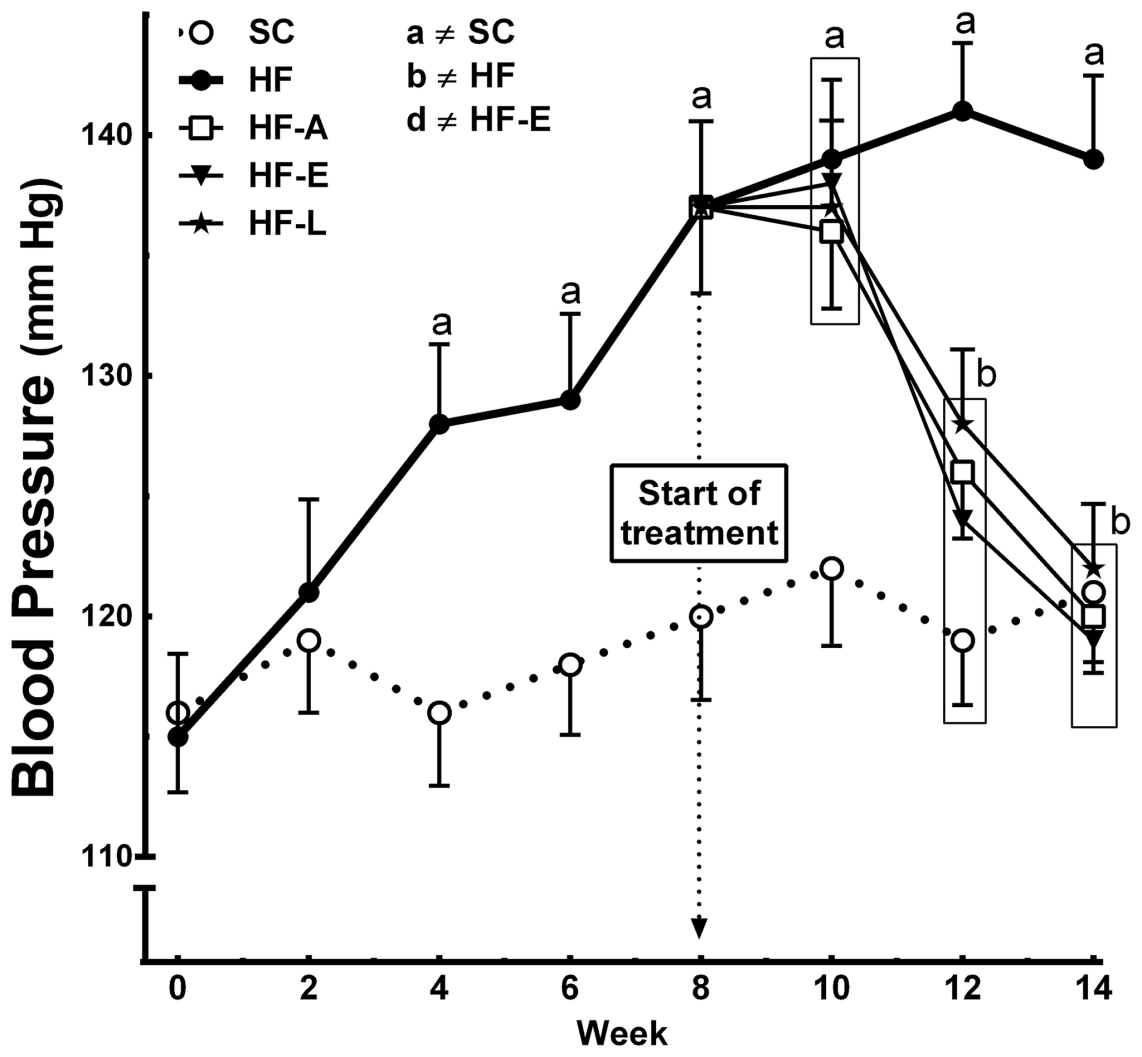
Systolic blood pressure evolution. Data are reported as the means ± SEM, n=8; *P*<0.05, one-way ANOVA and post-hoc of Holm-Sidak test: a ≠ SC; b ≠ HF; c ≠ HF-A, and d ≠ HF-E.

After four weeks of the diets, the BP was significantly increased in the animals fed HF diets, compared to that of the SC group (+10%, *P*<0.05). The difference continued to increase and reached 15% higher at 14 weeks (*P*<0.01) (Figure 2). All of the treatments that decreased BP had reached values similar to the SC group by the end of the experiment (Figure 2).

### Glucose and Insulin

**Figure 3:**
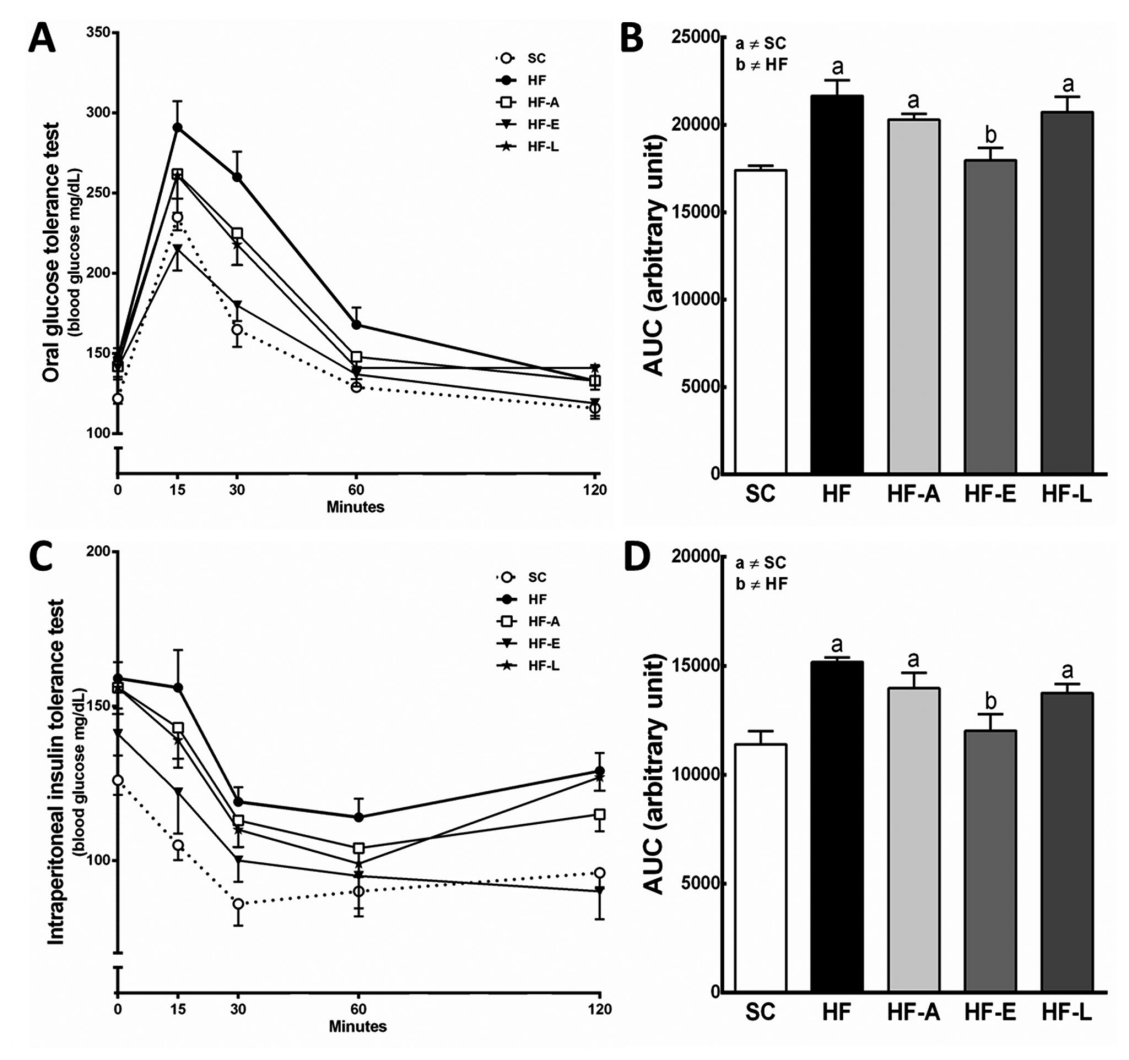
Glucose metabolism. Oral glucose tolerance test curves (A), and their respective area under the curves (AUC) (B); Intraperitoneal insulin tolerance test curves (C), and their respective AUC (D). Data are reported as the means ± SEM, n=8; *P*<0.05, one-way ANOVA and post-hoc of Holm-Sidak test: a ≠ SC; b ≠ HF.

The OGTT results were similar in both the SC and HF-E groups (Figure 3A and B). Compared to the SC group, the OGTT result was higher in the following groups: HF (+24%, *P*<0.01), HF-A (+17%, *P*<0.05), and HF-L (+19%, *P*<0.05) (Figure 3B). Compared to the HF group, enalapril ameliorated glucose tolerance, based on the lower levels of insulin necessary to clear the plasma glucose (-17%, *P*<0.01), the lower absolute fasting glucose levels, and the decreased rate of change after glucose overload administration in the first 15 to 30 minutes observed in the HF-E group (Figure 3A).

Compared to the SC group, the AUC total values to the IPITT were higher in the following groups: HF (+33%, *P*<0.01), HF-A (+23%, *P*<0.05), and HF-L (+21%, *P*<0.05) (Figure 3C and D). These results imply that these latter groups were less insulin sensitive, with consequent abnormally high glucose concentrations from 15 to 30 minutes compared with SC animals (Figure 3C). This is the typical feature of the islet dysfunction in the insulin resistance state. The HF-E group has shown a better insulin sensitivity in comparison to the HF group, since the AUC to the IPITT was smaller in the HF-E group than in the HF group (-21%, *P*<0.01) (Figure 3D).

### Metabolic parameters

Compared to the SC group, hyperinsulinemia and hyperglycemia were observed in the HF, HF-A, and HF-L groups (Table 1), which is consistent with the findings of glucose intolerance in the OGTT and insulin resistance in the IPITT (Figure 3). Consequently, the HF, HF-A, and HF-L groups showed both higher fasting plasma glucose and insulin levels and higher HOMA-IR compared to the SC group (*P*<0.01). However, enalapril markedly reduced the fasting plasma insulin (-27%, *P*<0.05) and glucose levels (-21%, *P*<0.001), resulting in a 48% lower HOMA-IR than the HF group (*P*<0.001) (Table 1).

Additionally, fasting plasma glucagon concentrations were higher in the HF (+126%; *P*< 0.001), HF-A (+75%; *P*<0.05), and HF-L (+108%; *P*<0.01) groups than in the SC group (Table 1), indicating that insulin resistance contributes to the dysregulation of glucagon secretion in altered glycemic states. The concentrations in the HF-E group was less elevated, and this difference was significant compared to the HF group (-43%; *P*<0.05) (Table 1).

Compared to the SC group, adiponectin levels were lower in the HF (-43%; *P*<0.0001), HF-A (-32%; *P*<0.0001), and HF-L (-26%; *P*<0.001) groups, but not in the HF-E group. Adiponectin levels were not different between the HF-E and SC groups, but the differences between the HF-E group and the other HF groups were significant: +71% compared to the HF group (*P*<0.0001), +44% compared to the HF-A group (*P*<0.0001), and +32% compared to the HF-L group (*P*<0.001) (Table 1). Adiponectin increases insulin sensitivity by several mechanisms, and these results are thus consistent with previous results regarding insulin resistance.

### Pancreas

**Figure 4:**
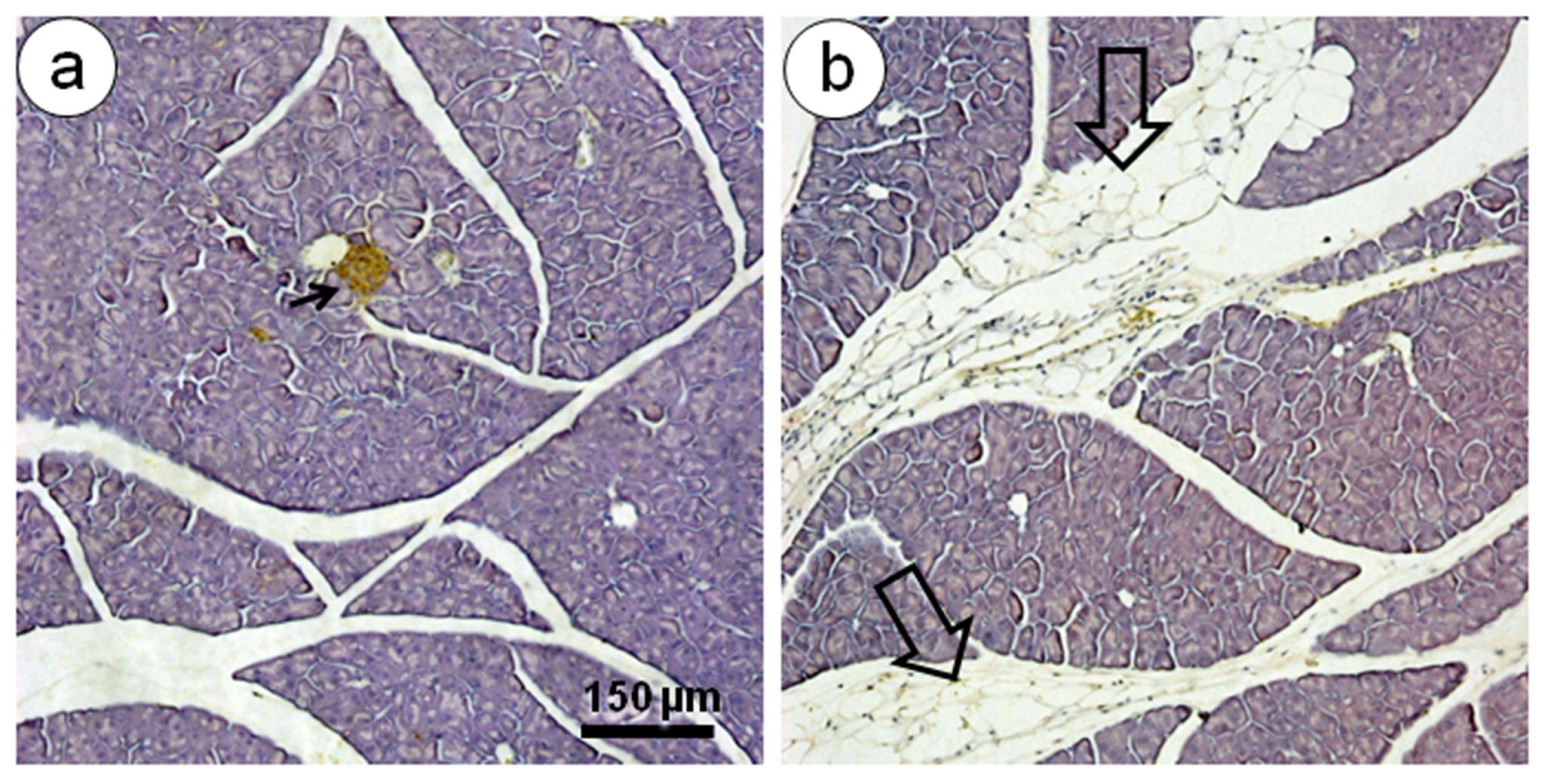
Pancreatic steatosis. Representative light micrographs of pancreatic sections (stain, hematoxylin and eosin; same magnification, bar = 150 µm) showing normal pancreatic tissues and a pancreatic islets (arrows) in the SC group (A). The HF diet group (B), with a large infiltration of fat (open arrows) between pancreatic lobules (interlobular fat) and within exocrine tissue (intralobular fat).

The islets were affected by the consumption of the HF diet (Table 2). All groups fed the HF diet, including the treated groups, showed an accumulation of fat within the pancreas in the interlobular, intralobular, and perilobular spaces. This accumulation was 230% greater in the HF group than in the SC group (*P*<0.0001) (Figure 4). In addition, the HF diet led to islet hypertrophy. Compared to the SC group, the islet mean diameter was 23% greater in the HF-E group (*P*<0.01). However, the islet mean diameter was 15% smaller in the HF-E group than in the HF group (*P*<0.01) (Table 2). The volume density of the islets correlated with the islet hypertrophy that was observed in the HF groups. Each of the HF, HF-A, and HF-L groups showed a greater volume density of islets than that of the SC group (*P*<0.01). However, the HF-E group recuperated the volume density of islets close to that of the SC group (Table 2).

**Table 2:** Pancreatic stereology, alpha and beta cell masses.

**Data**	**SC**	**HF**	**HF-A**	**HF-E**	**HF-L**
**Pancreas**					
Mass (mg)	278.3 ± 15.8	351.8 ± 25.1	318.5 ± 24.6	272.8 ± 18.2	340.5 ± 17.6
Fat (%)	3.7 ± 0.3	12.1 ± 1.5^[a]^	10.0 ± 0.6^[a]^	7.4 ± 0.3^[a,b]^	10.3 ± 0.5^[a]^
**Islet**					
Volume density (%)	10.5 ± 0.4	17.0 ± 0.7^[a]^	13.9 ± 0.7^[a,b]^	12.7 ± 0.5^[b]^	15.5 ± 0.5^[a,d]^
Mass (mg)	29.1 ± 1.6	57.1 ± 5.0^[a]^	46.2 ± 2.2^[a]^	34.4 ± 3.2^[b]^	57.7 ± 2.3^[a,d]^
Diameter (µm)	86.9 ± 3.9	125.6 ± 5.7^[a]^	111.5 ± 1.8^[a]^	106.8 ± 1.6^[a,b]^	119.8 ± 3.7^[a]^
**Alpha cell mass**					
Glucagon (%)	9.5 ± 0.3	15.3 ± 0.7^[a]^	12.7 ± 1.4^[a]^	10.5 ± 1.2^[b]^	12.7 ± 0.7^[a]^
Mass (mg)	2.8 ± 0.2	8.6 ± 0.5^[a]^	5.8 ± 0.5^[a,b]^	3.5 ± 0.2^[b,c]^	7.3 ± 0.3^[a,d]^
**Beta cell mass**					
Insulin (%)	70.3 ± 1.0	74.7 ± 1.0^[a^	72.4 ± 0.7	69.0 ± 1.6^[b]^	75.2 ± 1.4^[d]^
Mass (mg)	20.5 ± 1.2	42.7 ± 4.2^[a]^	33.5 ± 1.4^[a]^	23.6 ± 1.8^[b,c]^	41.2 ± 2.2^[a,d]^

Values represent means ± SEM, n=6 per group. Where indicated, P<0.05 using one-way ANOVA and post hoc Holm-Sidak test [a] when compared with the SC group; [b] when compared with the HF group; [c] when compared with the HF-A group and [d] when compared with the HF-E group.

The masses of alpha and beta cells in the islets were greater in the HF, HF-A, and HF-L groups than in the SC group (*P*<0.01). Only the HF-E group recuperated the masses of alpha and beta cells close to those of the SC group. Compared to the HF group, the alpha cell mass was 57% lower and the beta cell mass was 45% lower in the HF-E group (*P*<0.001 in both masses) (Table 2).

### Immunohistochemistry

**Figure 5:**
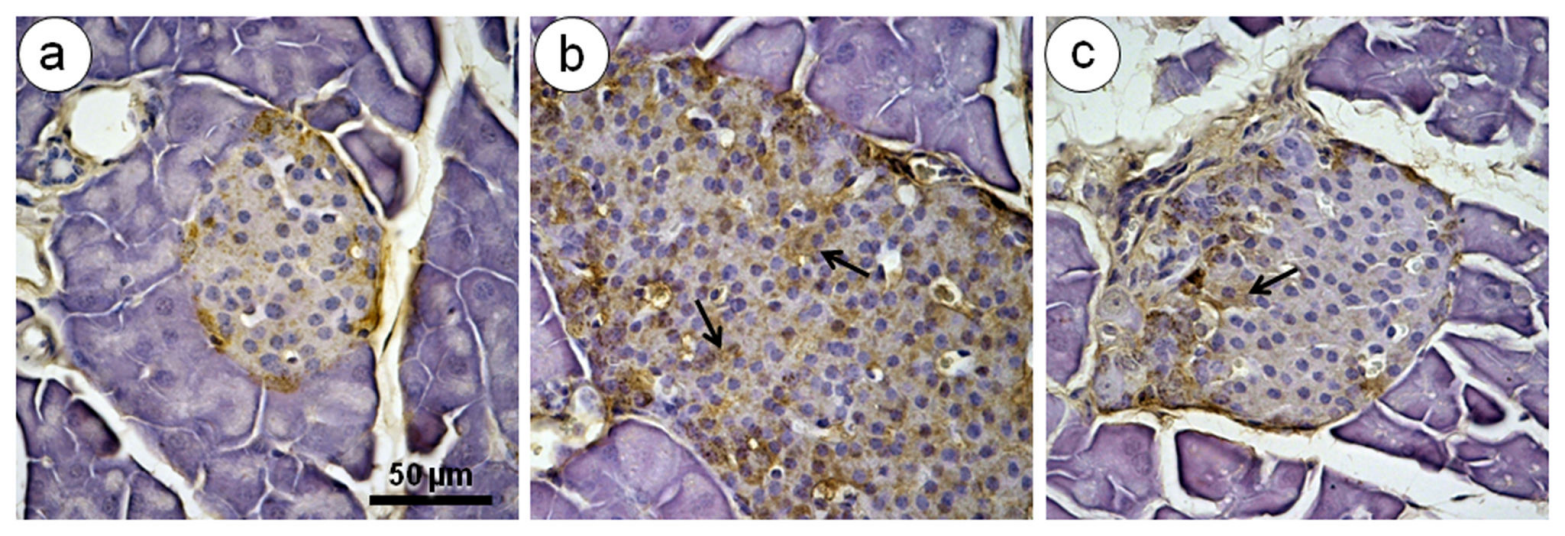
Alpha cells immunohistochemistry. Representative light micrographs of pancreatic sections stained with antibodies against glucagon (brown) and counterstained with hematoxylin (same magnification, bar = 50 µm). The untreated HF mice (B) display an increase in glucagon immunoreactive-positive alpha cells, and the presence of many alpha cells in both core and the periphery of the islet (arrows) implies an increased in alpha cell mass and a more intense secretion of glucagon, relative to the SC mice (A). The enalapril-treated mice (C) were similar to the SC mice and showed few infiltrations of alpha cells into the islet core (arrows).

**Figure 6:**
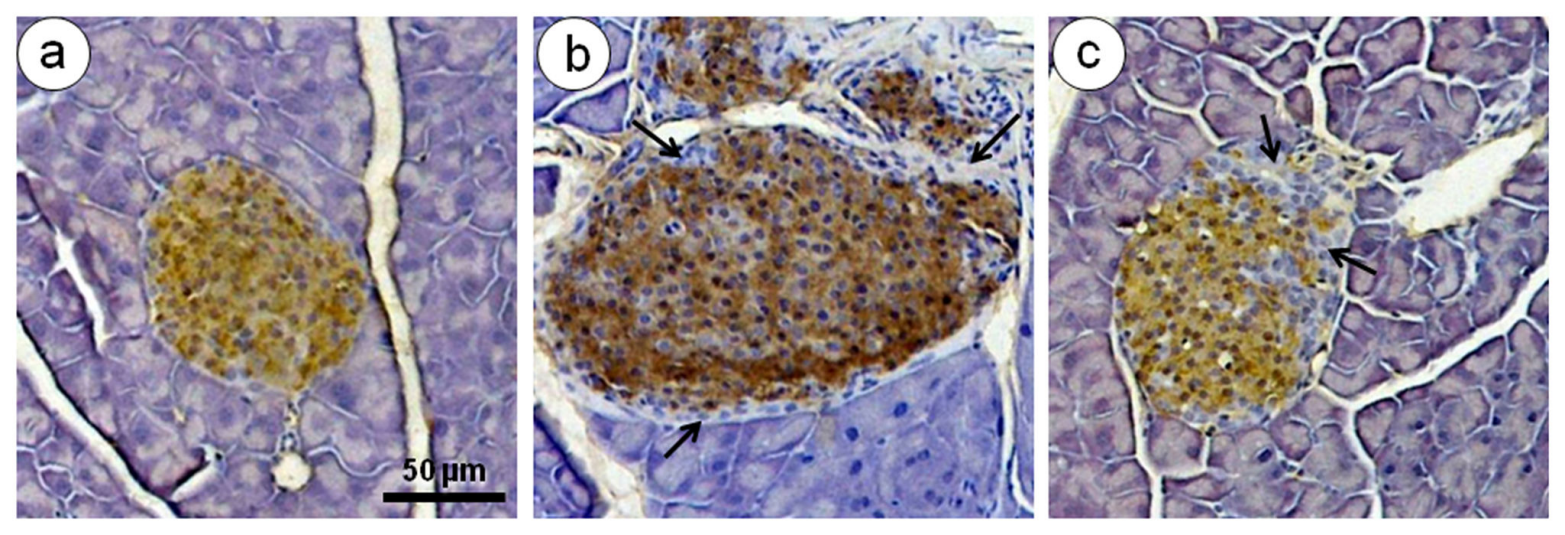
Beta cell immunohistochemistry. Photomicrographs show islets with immunoperoxidase (brown)-stained insulin (counterstained with hematoxylin, same magnification, bar = 50 µm). The SC group (A) shows smaller islets than the untreated HF group and a normal pattern of islet distribution, with beta cells restricted to the core of this structure. The untreated HF group (B) shows an increase in insulin immunoreactive-positive beta cells, representing an augmentation of beta cell mass, pancreatic islets hypertrophy, and a complete disarrangement of beta and non-beta cell distributions (arrows). Treatment with enalapril (C) displays a partial restoration of normal patterns of islet cell distributions (arrows) and islet size.

The SC group showed small islets with a regular pattern of islet distribution. In comparison to the SC group, the HF, HF-A, and HF-L groups showed increased glucagon (Figure 5A) and insulin (Figure 6A) immunoreactivity in alpha and beta cells, respectively. In these groups, the islets showed alpha cells in both the core and periphery and a thickening of the layer of non-beta cells, with disorganization in the distribution of the endocrine cells (Figure 5B). In addition, supporting the findings of insulin secretion and islet hypertrophy, the beta cell mass increased in the HF, HF-A, and HF-L groups (Table 2), and the disorganized histoarchitecture was consistent with the dysfunction of beta cells and insulin resistance (Figure 6B). The appearance of the alpha and beta cell distributions was similar between the SC group and the HF-E group (Figures 5C and 6C), which is consistent with the findings on the plasma insulin and glucagon levels in these groups.

### Western blotting analyses

The protein expressions in the HF groups were expressed as a percentage of the protein expression in the SC group. All Western blotting data were normalized to beta actin, which was used as a loading control. Beta actin expression did not differ among the SC, HF and HF-treated groups (data not shown).

#### Pdx1and GLUT2

**Figure 7:**
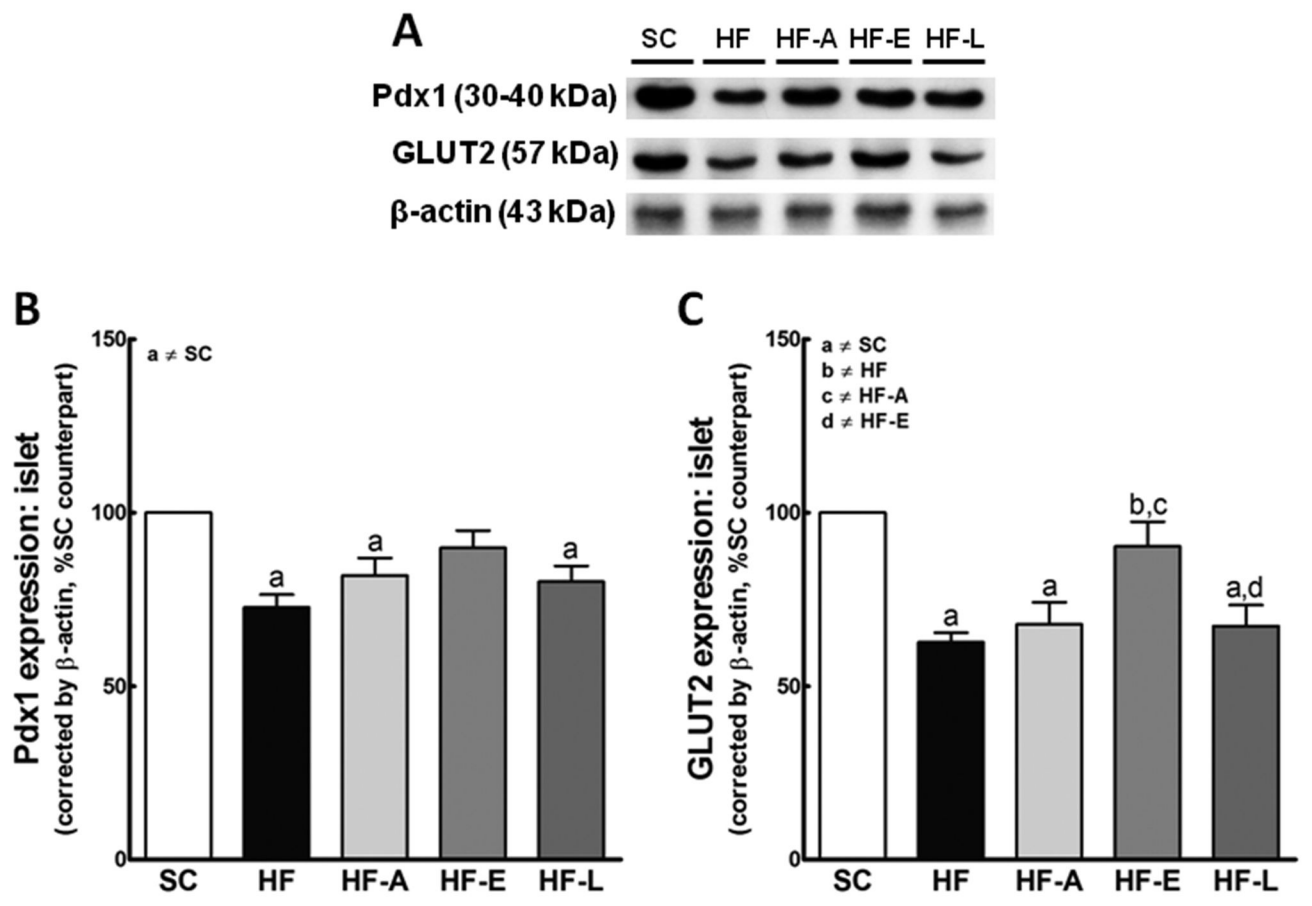
Representative Western blotting analysis of pancreatic islets for Pdx1 and GLUT2 expression (A) and their densitometry analysis (B – Pdx1; C – GLUT2). Average values were measured, and equal protein loading was confirmed by probing blots with beta actin antibodies. Each is, expressed as a percentage of the SC counterpart. Data are reported as the means ± SEM, n=9; *P*<0.05, one-way ANOVA and post-hoc of Holm-Sidak test: [a] compared to SC; [b] compared to untreated HF; [c] compared to HF-A, and [d] compared to HF-E.

The islet expression of Pdx1 and GLUT2 was lower in the HF group (*P*<0.001 and 0.0001, respectively), in the HF-A group (*P*<0.05 and 0.001, respectively), and in the HF-L group (*P*<0.05 and 0.001, respectively) than in the SC group (Figure 7). The Pdx1 and GLUT2 expression levels were not different between the HF-E and the SC groups.

#### RAS classical axis: Renin, ACE, AT1R and AT2R

**Figure 8:**
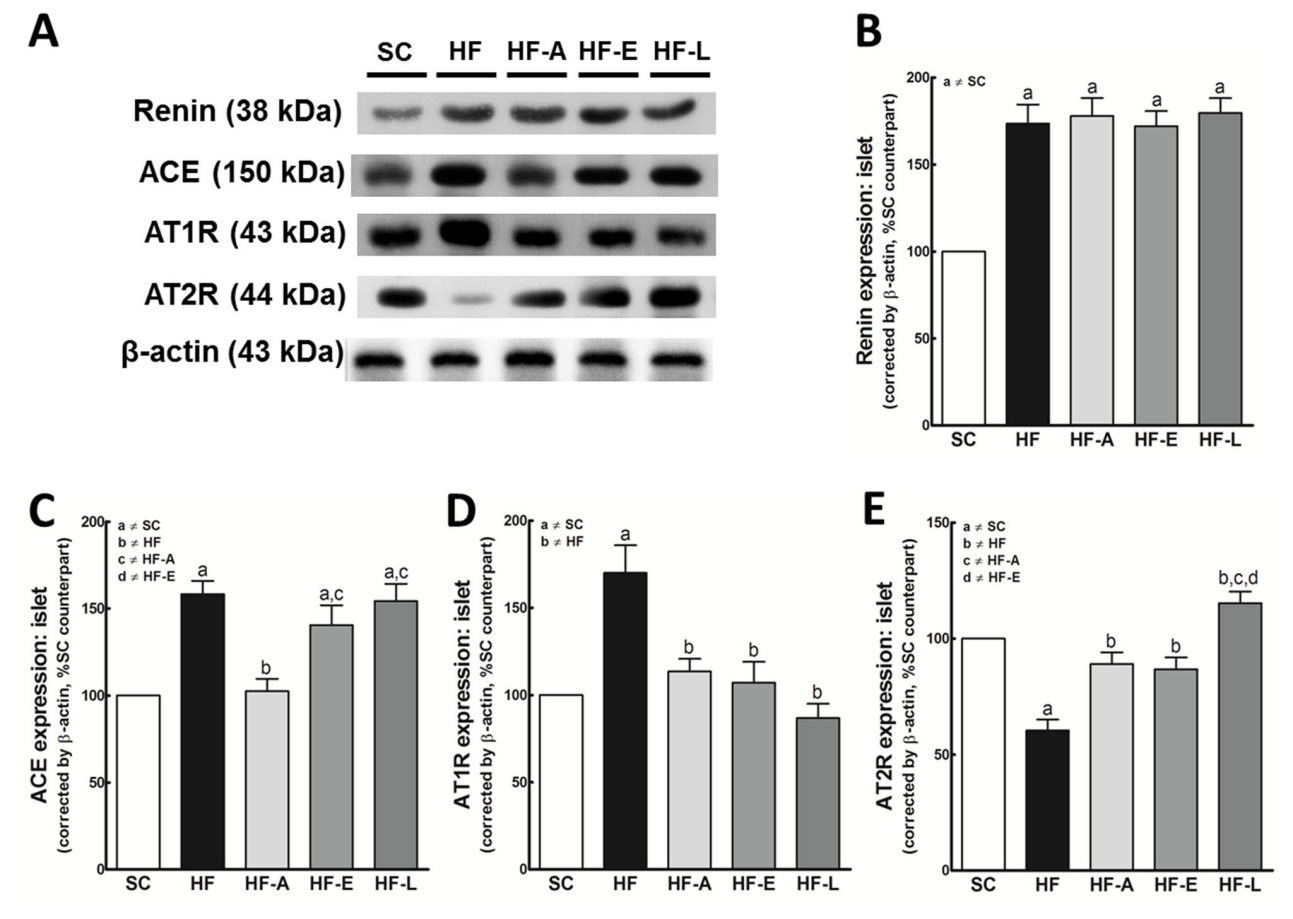
Western blotting analysis illustration (A) of pancreatic islets for renin (B), ACE (C), AT1R (D), and AT2R (E) expressions. Average values were measured, and equal protein loading was confirmed by probing blots with beta actin antibodies. Each is expressed as a percentage of the SC counterpart. Data are reported as the means ± SEM, n=9; *P*<0.05, one-way ANOVA and post-hoc of Holm-Sidak test: a ≠ SC; b ≠ HF; c ≠ HF-A and d ≠ HF-E.

Renin expression was higher in the treated groups, similar to the HF groups, than in the SC group (*P*<0.0001, in all cases) (Figure 8A and B). This result indicates that the islet RAS is up regulated in animal models of insulin resistance and obesity that are induced by diet.

ACE expression was lower in the HF-A group (*P*<0.01) compared to the HF group (Figure 8A and C). This result is most likely due to renin activity inhibition by aliskiren. However, ACE expression was higher in the HF, HF-E and HF-L groups compared to SC and HF-A groups (*P*<0.01) (Figure 8C).

AT1R expression in the treatment groups was similar to that of the SC group and significantly different from that of the HF group (*P*<0.01) (Figure 8A and D). Unlike AT1R, AT2R expression increased in all treated groups relative to the HF group (*P*<0.01); however, the peak levels were higher in the HF-L group (*P*<0.0001) (Figure 8A and E).

#### ACE2/*Mas* receptor axis

**Figure 9:**
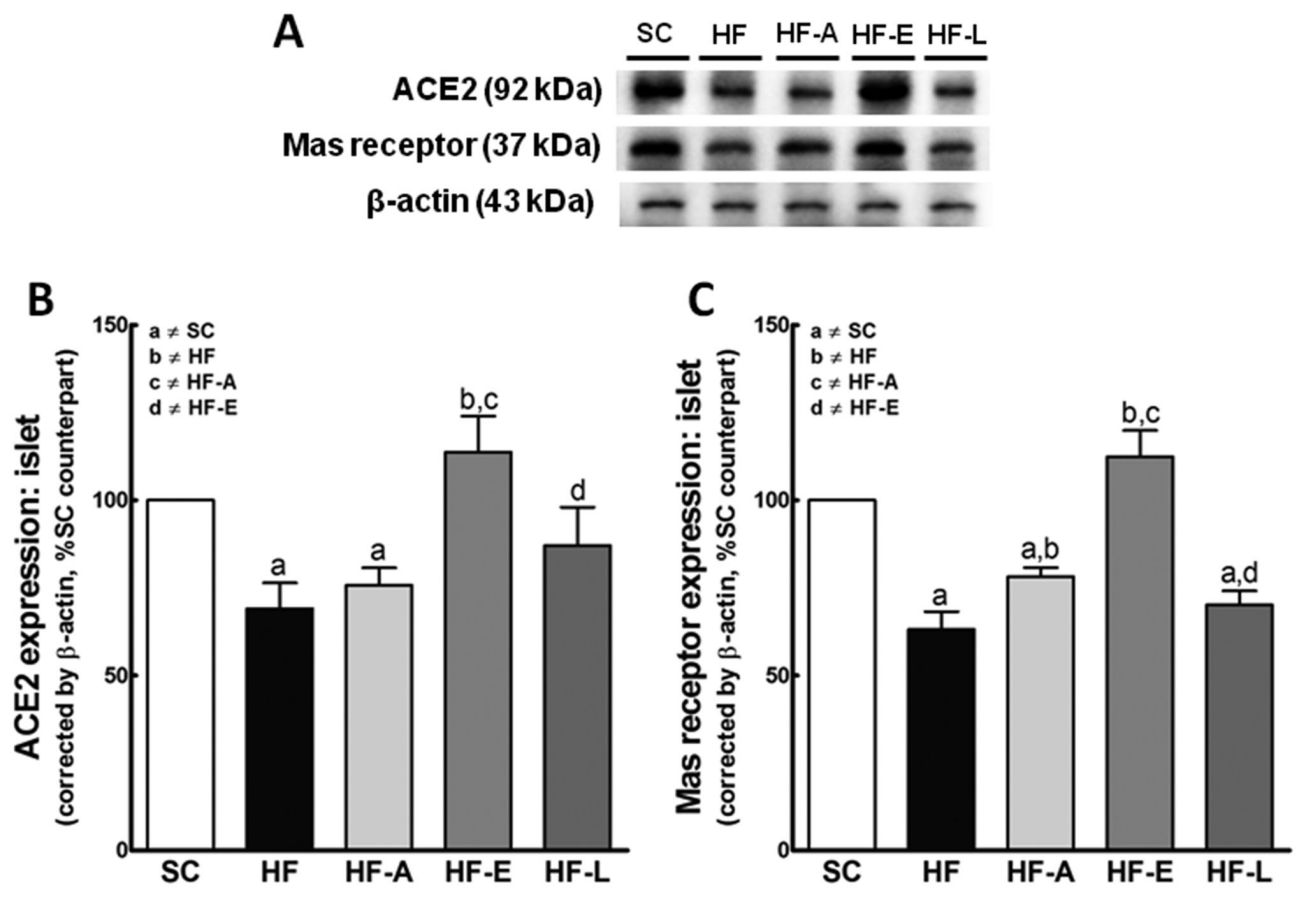
Western blotting analysis (A) of pancreatic islet for ACE2 (B), and *Mas* receptor (C) expression. Average values were measured, and equal protein loading was confirmed by probing blots with beta actin antibodies and is expressed as a percentage of the SC counterpart. Data are reported as the means ± SEM, n=9; *P*<0.05, one-way ANOVA and post-hoc of Holm-Sidak test: a ≠ SC; b ≠ HF; c ≠ HF-A and d ≠ HF-E.

The HF-A and HF-L groups had ACE2 expression levels similar to those of the HF group and lower than those of the SC group (*P*<0.01). Only treatment with enalapril caused an increase of ACE2 expression (*P*<0.0001 vs. HF group) (Figure 9A and B).

The *Mas* receptor expression was significantly lower in the HF, HF-A, and HF-L groups than in the SC group (*P*<0.01). In addition, *Mas* receptor expression was highest in the HF-E group (Figure 9A and C).

## Discussion

The present study compared RAS blockers, including a direct renin inhibitor, an ACE inhibitor and an AT1R antagonist, in a mice model of diet-induced obesity and insulin resistance. All treatments significantly attenuated the increased BP in HF mice. However, HF mice treated with enalapril, in addition to a reduction in both energy intake and BM, showed improved glucose tolerance and insulin sensitivity (as indicated by the improvement of fasting plasma insulin and glucose, as well as higher serum adiponectin), enhanced islet remodeling, normalization of both alpha and beta cell masses, and sustained beta cell function (Pdx1 and GLUT2 expression), compared to the HF group. Among the different mechanisms that may explain these findings, primarily the decreased energy intake and BM, the increase of the ACE2/ *Mas* receptor axis is a prime candidate. Our current results may contribute to understanding the role of systemic and local RAS in modulating Ang (1-7) and of the physiological effects of insulin in response to a HF diet.

The HF diet yielded overweight C57BL/6 mice with the highest BP and high expression levels of components of the RAS, among all the groups. In other words, the expression of renin, ACE and AT1R were increased in HF mice, proving the activation of classical RAS intra-islets. Ang II may be responsible for triggering inflammation by inducing oxidative stress [24], abnormalities of islet blood flow regulation, lipotoxicity and by negatively modulating insulin signaling [10], resulting in insulin resistance and endocrine pancreas dysfunction. In addition, HF mice showed the worst expression of the ACE2/*Mas* receptor axis. ACE2 is expressed in the liver, adipose tissue, pancreas and skeletal muscle, which constitute the primary organs implicated in insulin resistance [25,26] and exhibits its tissue-protective effects not only by decreasing Ang II but also by producing Ang (1-7). In fact, the genetic deletion of specific Ang (1-7) /*Mas* receptors leads to a metabolic syndrome-like state in mice, implying a close relationship between these axes and glucose metabolism [13,27].

There is substantial cross-talk between the insulin and Ang II signaling cascades, offering a potential mechanism for Ang II to impair insulin sensitivity [10]. Mice fed a HF diet have greater islet mass, accumulation of fat within the pancreas, hyperglycemia, and high plasma insulin and beta cell mass, resulting in a high HOMA-IR. There is mounting evidence that the acute exposure of pancreatic beta cells to high glucose concentrations results in a substantial increase in insulin release, whereas chronic exposure results in desensitization to secretions to the point of suppression [28]. This suppression contributes to a progressive decline in beta cell function that can lead to adverse pancreatic islet remodeling by processes commonly referred to as glucotoxicity, progressing into T2DM [29].

These events result in increased intra-islet insulin concentrations and the dysregulation of glucagon secretion, increasing alpha cell turnover and ultimately releasing more glucagon into circulation [30]. This is demonstrated by the rise in fasting plasma glucagon and alpha cell mass in the present study. Furthermore, we have observed hypertrophies and more irregularly shaped islets in the HF group. The disorganized histoarchitecture shown in this group is consistent with hyperinsulinemia and hyperglucagonemia and is characterized by alpha cell infiltration, a finding typically observed in diabetic animal models [31]. In addition, the lower levels of adiponectin observed in mice fed a HF diet, impairs insulin sensitivity through several mechanisms, including an increase in energy expenditure and fatty acid oxidation, an augmentation of the hepatic glucose output and a reduction in muscular glucose utilization [12].

This adverse islet remodeling is implicated in progressive beta cell dysfunction and determines the course of obesity-related insulin resistance. In the present study, HF mice had weak Pdx1 expression. In the mature pancreas, Pdx1 expression is linked to insulin-producing beta cells, gene transcription activation (glucose-stimulated insulin secretion) and sustained mature beta cell function. Preclinical studies demonstrated increased DNA methylation and decreased expression of Pdx1 in the development of T2DM [32]. In addition, Pdx1 also regulates the expression of GLUT2, both of which play a role in insulin resistance. In the present study, the HF diet reduced GLUT2 expression in hypertrophied islets. GLUT2 is a glucose sensor that triggers glucose-stimulated insulin secretion under physiological conditions in beta cells [33]; therefore, its decline indirectly suggests a worsening of beta cell function and insulin secretion.

The effectiveness of the doses administered to the treatment groups has been tested in previous studies in rodents (aliskiren [34], enalapril [35] and losartan [36]). Aliskiren administered to HF diet mice was efficient in reducing BP but did not significantly reverse the metabolic changes or adverse islet remodeling; it only partially enhanced the alpha cell mass and *Mas* receptor expression. As the initial limiting step in Ang II synthesis, renin represents a target for the complete inhibition of the RAS. However, the renin expression in islet tissue was increased by aliskiren, most likely due to inhibition of plasma renin activity [37]. In addition, renin inhibitors attenuate the production of all angiotensin derivatives from renin, inhibiting both their positive and negative effects on the metabolism. These findings suggest that the suppression of insulin resistance by aliskiren may require the use of higher doses than those used to treat high BP and cardiovascular injury [34], although aliskiren was efficient in renin inhibition in islets, as demonstrated by the observed reductions in ACE and AT1R levels.

Enalapril was the most successful treatment for the reduction of BM and insulin resistance in diet-induced obesity. The loss of body mass due to enalapril treatment is not an unexpected finding [38,39]. On the other hand, large randomized clinical trials suggest that ACE inhibitors improve insulin resistance and reduce the incidence of new-onset type 2 diabetes in high-risk patients with cardiovascular disease [40–43]. The data of the present study provide additional support for a role for the RAS in the control of energy balance and the potential for beneficial effects of ACE inhibitors as a therapeutic strategy for patients with obesity and concomitant hypertension. Furthermore, enalapril is most likely working through other pathways that modulate the reduction in BM gain (e.g., increased adiponectin that enhanced fatty acid oxidation, improved insulin and peroxisome proliferator-activated receptor (PPAR) gamma signaling, or increased expression of FAS) that corroborates with the decreased food intake [44,45].

Increased adiponectin levels have been associated with enhanced insulin sensitivity [45] and enalapril markedly attenuated the glucose and insulin resistance in mice in the HF group. We examined the effects of enalapril on endocrine pancreas morphology and function, including alpha and beta cell masses, the distribution of the cells into islets and Pdx1 and GLUT2 expressions. All of these parameters were improved in the mice of the HF-E group compared to the mice of the HF group. Enalapril significantly attenuated the increase of both alpha and beta cell masses, islet hypertrophy, and the islet cell disarray in the mice in the HF group. Additionally, enalapril significantly restored the Pdx1 and GLUT2 protein levels in the mice in the HF group, indicating the maintenance of beta cell function and glucose-stimulated insulin secretion. This result agrees with the idea that the ability of ACE inhibitors to augment insulin-stimulated glucose transport activity in insulin-resistant skeletal muscle is mediated primarily by the action of bradykinin, with little or no contribution from the decrease in Ang II action [46]. ACE inhibitors have at least two effects at the tissue level: inhibit the conversion of Ang I to Ang II and decreases the degradation of bradykinin [5]. It has been shown that the acute administration of captopril to insulin-resistant rats augmented the early steps of the insulin-signaling cascade [46] and this effects was reproduced by acute treatment with bradykinin, mediated through its B1 and B2 receptors [14], but not reproduced with losartan and eprosartan [5,46].

Our study shows that enalapril produces an increase in the expression of ACE2 and *Mas* receptors in the islet. There are two counter-regulatory peptides, Ang II and Ang (1-7), produced by the balance between ACE and ACE2 [47]. Therefore, a decreased ACE/ACE2 activity ratio by long-term treatment of enalapril stimulates generation of circulating Ang (1-7), and is followed by weight loss [48]. A lipolytic effect of Ang (1-7) has been recovered by administration of a *Mas* receptor blocker [47], and ACE-deficient mice had less body mass and fat than controls [49]. Additionally, ACE2 gene therapy improves glycemic control in diabetic mice by improving beta cell function through a mechanism mediated by Ang (1-7) [26]. It has been shown that an increase in circulating Ang (1-7) via a *Mas* receptor-dependent mechanism stimulates adiponectin release [50] and provides consequent beneficial effects on glucose utilization in peripheral tissues. These results provide evidence that the increased expression of the ACE2/*Mas* receptor axis by enalapril contributed to the above-mentioned protection against BM gain and the maintenance of the pancreatic islet functionality.

In the current study, losartan had no impact on body composition or food intake. However, any interpretations from studies on AT1R blockers should be considered carefully, as a number of AT1R blockers (e.g., irbesartan, telmisartan) are also partial PPAR gamma agonists; PPAR gamma plays an integral role in adipose differentiation and physiology [51]. Accordingly, our recent report suggested that telmisartan prevents BM gain and the normalization of islet morphology and function in mice fed a HF diet [51]. Only telmisartan activates PPAR gamma signaling, compared with losartan [52]. In addition, in the present study, losartan did not improve glucose homeostasis, adiponectin levels or pancreatic remodeling; instead, it normalized BP, as was also shown in obese hypertensive patients treated with losartan with impaired fasting glucose [53].

Animals treated with losartan had the highest AT2R expression in islets. In this way, the ability of Ang II to stimulate AT2R in the presence of and inhibitor of AT1R provided the additional activation of AT2R, generating a positive regulatory feedback. However, chronic exposure may cause receptor desensitization by high concentrations of Ang II. The role of AT2R stimulation in the pathogenesis of insulin resistance is still unclear [54]. Studies have shown that both AT1R and AT2R may modulate fat mass expansion through the upregulation of adipose tissue lipogenesis (AT2R) and the downregulation of lipolysis (AT1R) [55]. Additionally, AT2R deficient mice are protected against obesity that is induced by adipose tissue angiotensinogen overexpression, showing that AT2R plays a major role in mediating local Ang II action on fat mass enlargement [56]. Mice lacking AT2R fail to decrease adiponectin, increase whole-body lipid oxidation, or reduce their insulin resistance when fed a HF diet, indicating that AT2R-mediated Ang II signaling plays a crucial role in the control of energy metabolism and in glucose homeostasis [55].

The ACE2 and *Mas* receptors expression levels in islets were lower in HF-L mice than in either SC or HF-E mice. This agrees with reports that the reduced activity of the counter-regulatory ACE2/ Ang (1-7) /*Mas* axis results in glucose intolerance and reduced insulin sensitivity [57]. *Mas* receptor-deficient mice and/or mice with ACE2 ablation showed insulin resistance that was not eliminated by AT1R blockers but could be eradicated by Ang (1–7) [25,27]. Thus, losartan did not cause any changes in ACE2/ Ang (1-7) /*Mas* receptor axis [47]. Furthermore, ACE/ACE2 activity ratio increased due to increased Ang II generation and reduced generation of circulating Ang (1-7) that favored insulin resistance; an opposite ratio was found in enalapril treatment.

The effects of a RAS inhibitor on glucose homeostasis have been controversial and depend on the experimental model of obesity and insulin resistance, the time of starting the drug treatment, and differences in the dosage and duration of drug treatment. One of the limitations of the current study is that we did not measure plasma or tissue concentrations of Ang (1-7). However, this drawback does not exclude the results found by this work, as demonstrated by the G protein-coupled receptor expression, *Mas*, the specific receptor mediator of actions of Ang (1-7).

## Conclusion

The present study compared RAS blockers in mice fed HF diets and concluded that enalapril treatment greatly affected BM, energy intake and glucose tolerance, the normalization of the islet structure, both alpha cell and beta cell masses, and overall function (Pdx1 and GLUT2 expression). Thus, the current study provides evidence that enalapril protects the pancreatic islets against adverse remodeling in diet-induced obese mice with insulin resistance. The main findings include the following: (i) a reduction in food and energy intake that results in a small BM gain; (ii) an increase of adiponectin levels; (iii) an increase in the activity of the counter-regulatory ACE2/Ang (1-7) /*Mas* axis. In addition, losartan does not improve neither beta cell function nor insulin responsiveness in the HF mice, but increases the AT2R expression in islets. This indicates a role for AT2R in obesity-related disorders, principally regarding energy metabolism and insulin resistance. Further experimental and clinical studies are needed to clarify the precise mechanisms of RAS blockers, whether alone or in combination with other drugs, in cases of hypertension associated with T2DM. ACE inhibitors may be a promising therapeutic agent for obesity and its complications.

## References

[1] Richard D, Boisvert P (2009) 11th Annual International Symposium in Obesity: 'Obesity in a modern world: when pleasure meets homeostasis'. Int J Obes 33 Suppl 2: S1-S2. doi:10.1038/ijo.2008.259. 10.1038/ijo.2009.63 19528972

[2] Passos-Silva DG, Verano-Braga T, Santos RA (2013) Angiotensin-(1-7): beyond the cardio-renal actions. Clin Sci (Lond) 124: 443-456. doi:10.1042/CS20120461. 23249272 10.1042/CS20120461

[3] Luther JM, Brown NJ (2011) The renin-angiotensin-aldosterone system and glucose homeostasis. Trends Pharmacol Sci 32: 734-739. doi:10.1016/j.tips.2011.07.006. PubMed: 21880378. 21880378 10.1016/j.tips.2011.07.006 PMC3223326

[4] Peach MJ (1977) Renin-angiotensin system: biochemistry and mechanisms of action. Physiol Rev 57: 313-370. PubMed: 191856. 191856 10.1152/physrev.1977.57.2.313

[5] Henriksen EJ, Jacob S, Kinnick TR, Youngblood EB, Schmit MB et al. (1999) ACE inhibition and glucose transport in insulinresistant muscle: roles of bradykinin and nitric oxide. Am J Physiol Endocrinol Metab 277: R332-R336. 10.1152/ajpregu.1999.277.1.R332 10409290

[6] Donoghue M, Hsieh F, Baronas E, Godbout K, Gosselin M et al. (2000) A novel angiotensin-converting enzyme-related carboxypeptidase (ACE2) converts angiotensin I to angiotensin 1-9. Circ Res 87: E1-E9. doi:10.1161/01.RES.87.1.1. PubMed: 10969042. 10969042 10.1161/01.res.87.5.e1

[7] Giani JF, Mayer MA, Munoz MC, Silberman EA, Hocht C et al. (2009) Chronic infusion of angiotensin-(1-7) improves insulin resistance and hypertension induced by a high-fructose diet in rats. Am J Physiol Endocrinol Metab 296: E262-E271. PubMed: 19001546. 19001546 10.1152/ajpendo.90678.2008

[8] Santos RA, Simoes e Silva AC, Maric C, Silva DM, Machado RP et al. (2003) Angiotensin-(1-7) is an endogenous ligand for the G protein-coupled receptor Mas. PNAS 100: 8258-8263. doi:10.1073/pnas.1432869100. PubMed: 12829792. 12829792 10.1073/pnas.1432869100 PMC166216

[9] Leung PS (2003) Pancreatic renin-angiotensin system: a novel target for the potential treatment of pancreatic diseases? JOP 4: 89-91. PubMed: 12629265. 12629265

[10] Velloso LA, Folli F, Perego L, Saad MJ (2006) The multi-faceted cross-talk between the insulin and angiotensin II signaling systems. Diabetes/Metab Res Rev 22: 98-107. doi:10.1002/dmrr.611. 10.1002/dmrr.611 16389635

[11] van der Zijl NJ, Moors CC, Goossens GH, Blaak EE, Diamant M (2012) Does interference with the renin-angiotensin system protect against diabetes? Evidence and mechanisms. Diabetes Obes Metab 14: 586-595. doi:10.1111/j.1463-1326.2012.01559.x. PubMed: 22226145. 22226145 10.1111/j.1463-1326.2012.01559.x

[12] de Kloet AD, Krause EG, Woods SC (2010) The renin angiotensin system and the metabolic syndrome. Physiol Behav 100: 525-534. doi:10.1016/j.physbeh.2010.03.018. PubMed: 20381510. 20381510 10.1016/j.physbeh.2010.03.018 PMC2886177

[13] Munoz MC, Giani JF, Burghi V, Mayer MA, Carranza A et al. (2012) The Mas receptor mediates modulation of insulin signaling by angiotensin-(1-7). Regulat Peptides 177: 1-11. 10.1016/j.regpep.2012.04.001 22561450

[14] Barros CC, Haro A, Russo FJ, Schadock I, Almeida SS et al. (2012) Altered glucose homeostasis and hepatic function in obese mice deficient for both kinin receptor genes. PLOS ONE 7: e40573. doi:10.1371/journal.pone.0040573. PubMed: 22829877. 22829877 10.1371/journal.pone.0040573 PMC3400662

[15] Babu DA, Deering TG, Mirmira RG (2007) A feat of metabolic proportions: Pdx1 orchestrates islet development and function in the maintenance of glucose homeostasis. Mol Genet Metab 92: 43-55. doi:10.1016/j.ymgme.2007.06.008. PubMed: 17659992. 17659992 10.1016/j.ymgme.2007.06.008 PMC2042521

[16] Bernardo AS, Hay CW, Docherty K (2008) Pancreatic transcription factors and their role in the birth, life and survival of the pancreatic beta cell. Mol Cell Endocrinol 294: 1-9. doi:10.1016/j.mce.2008.07.006. PubMed: 18687378. 18687378 10.1016/j.mce.2008.07.006

[17] Navarro-Tableros V, Fiordelisio T, Hernandez-Cruz A, Hiriart M (2007) Physiological development of insulin secretion, calcium channels, and GLUT2 expression of pancreatic rat beta-cells. Am J Physiol Endocrinol Metab 292: E1018-E1029. PubMed: 17148757. 17148757 10.1152/ajpendo.00457.2006

[18] Reeves PG, Nielsen FH, Fahey GC Jr. (1993) AIN-93 purified diets for laboratory rodents: final report of the American Institute of Nutrition ad hoc writing committee on the reformulation of the AIN-76A rodent diet. J Nutr 123: 1939-1951. PubMed: 8229312. 8229312 10.1093/jn/123.11.1939

[19] Matthews DR, Hosker JP, Rudenski AS, Naylor BA, Treacher DF et al. (1985) Homeostasis model assessment: insulin resistance and beta-cell function from fasting plasma glucose and insulin concentrations in man. Diabetologia 28: 412-419. doi:10.1007/BF00280883. PubMed: 3899825. 3899825 10.1007/BF00280883

[20] Mandarim-de-Lacerda CA, Fernandes-Santos C, Aguila MB (2010) Image analysis and quantitative morphology. Methods Mol Biol 611: 211-225. doi:10.1007/978-1-60327-345-9_17. PubMed: 19960334. 19960334 10.1007/978-1-60327-345-9_17

[21] Tschanz SA, Burri PH, Weibel ER (2011) A simple tool for stereological assessment of digital images: the STEPanizer. J Microsc 243: 47-59. doi:10.1111/j.1365-2818.2010.03481.x. PubMed: 21375529. 21375529 10.1111/j.1365-2818.2010.03481.x

[22] Frantz ED, Aguila MB, Pinheiro-Mulder Ada R, Mandarim-de-Lacerda CA (2011) Transgenerational endocrine pancreatic adaptation in mice from maternal protein restriction in utero. Mech Ageing Dev 132: 110-116. 21291904 10.1016/j.mad.2011.01.003

[23] Lacy PE, Kostianovsky M (1967) Method for the isolation of intact islets of Langerhans from the rat pancreas. Diabetes 16: 35-39. PubMed: 5333500. 5333500 10.2337/diab.16.1.35

[24] Yuan L, Li X, Xu GL, Qi CJ (2010) Effects of renin-angiotensin system blockade on islet function in diabetic rats. J Endocrinol Invest 33: 13-19. 10.1007/BF03346544 20203537

[25] Takeda M, Yamamoto K, Takemura Y, Takeshita H, Hongyo K et al. (2012) Loss of ACE 2 Exaggerates High-Calorie Diet-Induced Insulin Resistance by Reduction of GLUT4 in Mice. Diabetes 62: 223-233. PubMed: 22933108. 22933108 10.2337/db12-0177 PMC3526031

[26] Bindom SM, Hans CP, Xia H, Boulares AH, Lazartigues E (2010) Angiotensin I-converting enzyme type 2 (ACE2) gene therapy improves glycemic control in diabetic mice. Diabetes 59: 2540-2548. doi:10.2337/db09-0782. PubMed: 20660625. 20660625 10.2337/db09-0782 PMC3279528

[27] Santos SH, Fernandes LR, Mario EG, Ferreira AV, Porto LC et al. (2008) Mas deficiency in FVB/N mice produces marked changes in lipid and glycemic metabolism. Diabetes 57: 340-347. PubMed: 18025412. 18025412 10.2337/db07-0953

[28] Lowell BB, Shulman GI (2005) Mitochondrial dysfunction and type 2 diabetes. Science 307: 384-387. doi:10.1126/science.1104343. PubMed: 15662004. 15662004 10.1126/science.1104343

[29] Bonora E (2008) Protection of pancreatic beta-cells: is it feasible? Nutr Metab Cardiovasc Dis 18: 74-83. doi:10.1016/j.numecd.2007.05.004. PubMed: 18096375. 18096375 10.1016/j.numecd.2007.05.004

[30] Liu Z, Kim W, Chen Z, Shin YK, Carlson OD et al. (2011) Insulin and glucagon regulate pancreatic alpha-cell proliferation. PLOS ONE 6: e16096. doi:10.1371/journal.pone.0016096. PubMed: 21283589. 21283589 10.1371/journal.pone.0016096 PMC3026810

[31] Janssen SW, Hermus AR, Lange WP, Knijnenburg Q, van der Laak JA et al. (2001) Progressive histopathological changes in pancreatic islets of Zucker Diabetic Fatty rats. Exp Clin Endocrinol Diabetes 109: 273-282. doi:10.1055/s-2001-16347. PubMed: 11507651. 11507651 10.1055/s-2001-16347

[32] Yang BT, Dayeh TA, Volkov PA, Kirkpatrick CL, Malmgren S et al. (2012) Increased DNA methylation and decreased expression of PDX-1 in pancreatic islets from patients with type 2 diabetes. Mol Endocrinol 26: 1203-1212. doi:10.1210/me.2012-1004. PubMed: 22570331. 22570331 10.1210/me.2012-1004 PMC5416998

[33] Folli F, Okada T, Perego C, Gunton J, Liew CW et al. (2011) Altered insulin receptor signalling and beta-cell cycle dynamics in type 2 diabetes mellitus. PLOS ONE 6: e28050. doi:10.1371/journal.pone.0028050. PubMed: 22140505. 22140505 10.1371/journal.pone.0028050 PMC3227614

[34] Dong YF, Liu L, Kataoka K, Nakamura T, Fukuda M et al. (2010) Aliskiren prevents cardiovascular complications and pancreatic injury in a mouse model of obesity and type 2 diabetes. Diabetologia 53: 180-191. doi:10.1007/s00125-009-1575-5. PubMed: 19894030. 19894030 10.1007/s00125-009-1575-5

[35] Pereira LM, Bezerra DG, Machado DL, Mandarim-de-Lacerda CA (2004) Enalapril attenuates cardiorenal damage in nitric-oxide-deficient spontaneously hypertensive rats. Clin Sci (Lond) 106: 337-343. doi:10.1042/CS20030268. 14629192 10.1042/CS20030268

[36] Chu KY, Lau T, Carlsson PO, Leung PS (2006) Angiotensin II type 1 receptor blockade improves beta-cell function and glucose tolerance in a mouse model of type 2 diabetes. Diabetes 55: 367-374. doi:10.2337/diabetes.55.02.06.db05-1022. PubMed: 16443769. 16443769 10.2337/diabetes.55.02.06.db05-1022

[37] Staessen JA, Li Y, Richart T (2006) Oral renin inhibitors. Lancet 368: 1449-1456. doi:10.1016/S0140-6736(06)69442-7. PubMed: 17055947. 17055947 10.1016/S0140-6736(06)69442-7

[38] Masuo K, Mikami H, Ogihara T, Tuck ML (2001) Weight reduction and pharmacologic treatment in obese hypertensives. Am J Hypertens 14: 530-538. doi:10.1016/S0895-7061(00)01279-6. PubMed: 11411732. 11411732 10.1016/s0895-7061(00)01279-6

[39] Santos EL, de Picoli Souza K, da Silva ED, Batista EC, Martins PJ et al. (2009) Long term treatment with ACE inhibitor enalapril decreases body weight gain and increases life span in rats. Biochem Pharmacol 78: 951-958. doi:10.1016/j.bcp.2009.06.018. PubMed: 19549507. 19549507 10.1016/j.bcp.2009.06.018

[40] Barzilay JI, Davis BR, Cutler JA, Pressel SL, Whelton PK et al. (2006) Fasting glucose levels and incident diabetes mellitus in older nondiabetic adults randomized to receive 3 different classes of antihypertensive treatment: a report from the Antihypertensive and Lipid-Lowering Treatment to Prevent Heart Attack Trial (ALLHAT). Arch Intern Med 166: 2191-2201. doi:10.1001/archinte.166.20.2191. PubMed: 17101936. 17101936 10.1001/archinte.166.20.2191

[41] Yusuf S, Sleight P, Pogue J, Bosch J, Davies R et al. (2000) Effects of an angiotensin-converting-enzyme inhibitor, ramipril, on cardiovascular events in high-risk patients. The Heart Outcomes Prevention Evaluation Study Investigators. NEJM 342: 145-153. doi:10.1056/NEJM200001203420301. PubMed: 10639539. 10639539 10.1056/NEJM200001203420301

[42] Hansson L, Lindholm LH, Niskanen L, Lanke J, Hedner T et al. (1999) Effect of angiotensin-converting-enzyme inhibition compared with conventional therapy on cardiovascular morbidity and mortality in hypertension: the Captopril Prevention Project (CAPPP) randomised trial. Lancet 353: 611-616. doi:10.1016/S0140-6736(98)05012-0. PubMed: 10030325. 10030325 10.1016/s0140-6736(98)05012-0

[43] Shindler DM, Kostis JB, Yusuf S, Quinones MA, Pitt B et al. (1996) Diabetes mellitus, a predictor of morbidity and mortality in the Studies of Left Ventricular Dysfunction (SOLVD) Trials and Registry. Am J Cardiol 77: 1017-1020. doi:10.1016/S0002-9149(97)89163-1. PubMed: 8644628. 8644628 10.1016/s0002-9149(97)89163-1

[44] Santos EL, de Picoli Souza K, da Silva ED, Batista EC, Martins PJ et al. (2009) Long term treatment with ACE inhibitor enalapril decreases body weight gain and increases life span in rats. Biochem Pharmacol 78: 951-958. doi:10.1016/j.bcp.2009.06.018. PubMed: 19549507. 19549507 10.1016/j.bcp.2009.06.018

[45] Weisinger RS, Stanley TK, Begg DP, Weisinger HS, Spark KJ et al. (2009) Angiotensin converting enzyme inhibition lowers body weight and improves glucose tolerance in C57BL/6J mice maintained on a high fat diet. Physiol Behav 98: 192-197. doi:10.1016/j.physbeh.2009.05.009. PubMed: 19465040. 19465040 10.1016/j.physbeh.2009.05.009

[46] Carvalho CR, Thirone AC, Gontijo JA, Velloso LA, Saad MJ (1997) Effect of captopril, losartan, and bradykinin on early steps of insulin action. Diabetes 46: 1950-1957. doi:10.2337/diabetes.46.12.1950. PubMed: 9392479. 9392479 10.2337/diab.46.12.1950

[47] Oh YB, Kim JH, Park BM, Park BH, Kim SH (2012) Captopril intake decreases body weight gain via angiotensin-(1-7). Peptides 37: 79-85. doi:10.1016/j.peptides.2012.06.005. PubMed: 22743141. 22743141 10.1016/j.peptides.2012.06.005

[48] Santos RA, Ferreira AJ, Esac Simoes (2008) Recent advances in the angiotensin-converting enzyme 2-angiotensin(1-7)-Mas axis. Exp Physiol 93: 519-527. doi:10.1113/expphysiol.2008.042002. PubMed: 18310257. 18310257 10.1113/expphysiol.2008.042002

[49] Jayasooriya AP, Mathai ML, Walker LL, Begg DP, Denton DA et al. (2008) Mice lacking angiotensin-converting enzyme have increased energy expenditure, with reduced fat mass and improved glucose clearance. PNAS 105: 6531-6536. doi:10.1073/pnas.0802690105. PubMed: 18443281. 18443281 10.1073/pnas.0802690105 PMC2373349

[50] Santos SH, Braga JF, Mario EG, Porto LC, Rodrigues-Machado MG et al. (2010) Improved lipid and glucose metabolism in transgenic rats with increased circulating angiotensin-(1-7). Arterioscler Thromb Vasc Biol 30: 953-961. doi:10.1161/ATVBAHA.109.200493. PubMed: 20203301. 20203301 10.1161/ATVBAHA.109.200493

[51] Souza-Mello V, Gregorio BM, Cardoso-de-Lemos FS, de Carvalho L, Aguila MB et al. (2010) Comparative effects of telmisartan, sitagliptin and metformin alone or in combination on obesity, insulin resistance, and liver and pancreas remodelling in C57BL/6 mice fed on a very high-fat diet. Clin Sci (Lond) 119: 239-250. doi:10.1042/CS20100061. 20415664 10.1042/CS20100061

[52] Kintscher U (2012) And in the end -- Telmisartan directly binds to PPARgamma. Hypert Res 35: 704-705. 10.1038/hr.2012.35 22437039

[53] Perlstein TS, Henry RR, Mather KJ, Rickels MR, Abate NI et al. (2012) Effect of angiotensin receptor blockade on insulin sensitivity and endothelial function in abdominally obese hypertensive patients with impaired fasting glucose. Clin Sci (Lond) 122: 193-202. 21861845 10.1042/CS20110284 PMC4566948

[54] Ohshima K, Mogi M, Jing F, Iwanami J, Tsukuda K et al. (2012) Direct Angiotensin II Type 2 Receptor Stimulation Ameliorates Insulin Resistance in Type 2 Diabetes Mice with PPARgamma Activation. PLOS ONE 7: e48387. doi:10.1371/journal.pone.0048387. PubMed: 23155382. 23155382 10.1371/journal.pone.0048387 PMC3498306

[55] Yvan-Charvet L, Even P, Bloch-Faure M, Guerre-Millo M, Moustaid-Moussa N et al. (2005) Deletion of the angiotensin type 2 receptor (AT2R) reduces adipose cell size and protects from diet-induced obesity and insulin resistance. Diabetes 54: 991-999. doi:10.2337/diabetes.54.4.991. PubMed: 15793237. 15793237 10.2337/diabetes.54.4.991

[56] Yvan-Charvet L, Quignard-Boulange A (2011) Role of adipose tissue renin-angiotensin system in metabolic and inflammatory diseases associated with obesity. Kidney Int 79: 162-168. doi:10.1038/ki.2010.391. PubMed: 20944545. 20944545 10.1038/ki.2010.391

[57] Mario EG, Santos SH, Ferreira AV, Bader M, Santos RA et al. (2012) Angiotensin-(1-7) Mas-receptor deficiency decreases peroxisome proliferator-activated receptor gamma expression in adipocytes. Peptides 33: 174-177. doi:10.1016/j.peptides.2011.11.014. PubMed: 22119778. 22119778 10.1016/j.peptides.2011.11.014

